# An overview of generic tools for information-theoretic secrecy performance analysis over wiretap fading channels

**DOI:** 10.1186/s13638-021-02065-4

**Published:** 2021-12-04

**Authors:** Long Kong, Yun Ai, Lei Lei, Georges Kaddoum, Symeon Chatzinotas, Björn Ottersten

**Affiliations:** 1 Interdisciplinary Centre for Security, Reliability and Trust (SnT), University of Luxembourg, Luxembourg City, 1855 Luxembourg; 2 Faculty of Engineering, Norwegian University of Science and Technology, Gjøvik, 2815 Norway; 3 School of Information and Communications Engineering, Xi’an Jiaotong University, Xi’an, 710049 China; 4 LaCIME Laboratory, Department of Electrical Engineering, École de Technologie Supérieure (ÉTS), Université du Québec, Montréal, QC H3C 1K3 Canada

**Keywords:** Physical layer security (PLS), Channel state information (CSI), Mixture Gamma (MG), Mixture of Gaussian (MoG), Fox’s *H*-function, Artificial noise (AN), Artificial fast fading (AFF), Wiretap fading model, Jamming, Antenna selection

## Abstract

Physical layer security (PLS) has been proposed to afford an extra layer of security on top of the conventional cryptographic techniques. Unlike the conventional complexity-based cryptographic techniques at the upper layers, physical layer security exploits the characteristics of wireless channels, e.g., fading, noise, interference, etc., to enhance wireless security. It is proved that secure transmission can benefit from fading channels. Accordingly, numerous researchers have explored what fading can offer for physical layer security, especially the investigation of physical layer security over wiretap fading channels. Therefore, this paper aims at reviewing the existing and ongoing research works on this topic. More specifically, we present a classification of research works in terms of the four categories of fading models: (i) small-scale, (ii) large-scale, (iii) composite, and (iv) cascaded. To elaborate these fading models with a generic and flexible tool, three promising candidates, including the mixture gamma (MG), mixture of Gaussian (MoG), and Fox’s *H*-function distributions, are comprehensively examined and compared. Their advantages and limitations are further demonstrated via security performance metrics, which are designed as vivid indicators to measure how perfect secrecy is ensured. Two clusters of secrecy metrics, namely (i) secrecy outage probability (SOP), and the lower bound of SOP; and (ii) the probability of nonzero secrecy capacity (PNZ), the intercept probability, average secrecy capacity (ASC), and ergodic secrecy capacity, are displayed and, respectively, deployed in passive and active eavesdropping scenarios. Apart from those, revisiting the secrecy enhancement techniques based on Wyner’s wiretap model, the on-off transmission scheme, jamming approach, antenna selection, and security region are discussed.

## Introduction

As stated in the latest released statistics by the International Telecommunications Union (ITU) in 2020 [1], COVID-19, to some extent, acts as an accelerator that pushes consumers and businesses to largely adopt digital services and technologies, which in return quickens the digital transformation for societies, business, and governments. Examples, including online learning, digital classrooms, contactless payment, zoom meetings, etc., are reshaping everyone’s life pattern. In light of the highly confidential data streams flowing over the wireless transmission medium, the legitimate data transactions enjoy the convenience largely brought by the inherent openness of the wireless transmission medium while facing the vulnerability of being exposed to illegitimate evil parties.

Traditionally, cryptography is an appealing approach to achieve data confidentiality. It is designed to prevent data disclosure to unauthorized devices and malicious users [2]. Although secrecy is guaranteed through the key-based encoding and decoding process and requires additional computing resources, it in fact assumes there exist error-free links at the physical layer. Such an assumption would be unfeasible for the emerging decentralized networks (e.g., resource-limited sensors or radio-frequency identification (RFID) networks) due to the high computational complexity and necessary key distribution and management [3]. Besides, the impacts from the impairments of wireless transmission medium on physical layer security, i.e., the randomness of wireless channels, are totally ignorant in cryptography.

Unlike the conventional complexity-based cryptographic techniques at upper layers via encryption, physical layer security (PLS), being a promising technology complementary to cryptography and certainly not as a replacement, takes full advantage of the physical properties of the wireless propagation environment via the combination of signaling and coding mechanism to provide additional secrecy at the bottom layer [4, 5]. It is proved suitable and feasible for achieving information-theoretic security against eavesdropping attacks. More specifically, under the cover of the randomness of noise, fading, and interference, different users will receive different noisy copies of the private messages. This can enable the confidentiality of legitimate transmissions at the physical layer.

As a promising approach, physical layer security is built on the two pioneering works laid by Shannon [6] and Wyner [7], where the notion of perfect secrecy and the degraded wiretap channel model are introduced, respectively. It is noteworthy to point out that Wyner’s result established the PLS from the system model level, and he considered the three-user scenario, consisting of a legitimate source (Alice), an intended legitimate user (Bob), and an eavesdropper (Eve) over the discrete memoryless wiretap channel. In [8], Wyner’s wiretap model was extended to the Gaussian wiretap channel by Leung et al., and they found the fundamental basis of secrecy capacity ($C_s$), which is defined as the difference between the channel capacity of the main channel (Alice to Bob, i.e., $C_M$) and that of the wiretap channel (Alice to Eve, i.e., $C_W$), namely, $C_s = C_M - C_W$. The conceptual implication of secrecy capacity indicates that only when the legitimate link experiences better quality of received signals compared to the wiretap channel, positive secrecy can be surely guaranteed. Inspired by this fundamental work, considerable research efforts have been devoted to investigate the security performance metrics over wiretap fading channels, e.g., [9, 10]. The insights drawn from these works offer mathematical proofs showing that wireless channels’ fading property can be reversely used to enhance secrecy.

Observing the existing books, surveys, and tutorials related to the PLS [2–5, 11–30], numerous researchers from both the wireless communication and signal processing communities summarized the state-of-the-art of PLS from the perspective of application scenarios, e.g., 5G wireless networks [25], cooperative networks [26], and ultra-reliable and low-latency communications (URLLC) [27], and secrecy enhancement, including jamming schemes [3, 19, 26], multiple-antenna techniques [24], and wiretap coding [14, Chapter 6] [25] (e.g., low-density parity-check (LDPC) codes, polar codes, and lattice codes.) . It is reported in [2] that Zou et al. have classified the PLS technique into four categories: information-theoretic security, artificial-noise aided security, security-oriented beamforming, security diversity methods, and physical layer secret key generation.

As an indispensable element of PLS techniques, information-theoretic security has been further classified into three categories according to different wiretap channels: (i) memoryless wiretap channels; (ii) Gaussian wiretap channels; and (iii) fading wiretap channels. However, the majority of information-theoretic security is centered around the fading wiretap channels, e.g., see references [9, 10, 31]. The pioneering work is laid by Bloch et al. [9], where the authors explored the impacts of fading characteristic of wireless channels on the security issue and proposed two performance metrics, i.e., the average secrecy capacity (ASC) and outage probability of secrecy capacity (equivalently, secrecy outage probability (SOP)), to measure information-theoretic security. At the same year, Gopala et al. [10] investigated the perfect secrecy capacity over wiretap fading channels for two scenarios: (i) the full channel state information (CSI) is available at the transmitter; and (ii) only the main channel CSI is perfectly known at the transmitter. The former scenario represents the active eavesdropping, to be specific, Eve is a legitimate network participant (e.g., in a time-division multiple-access (TDMA) environment). As a result, Alice is capable of accessing Eve’s CSI, as well as Bob’s CSI. Alice can adapt her coding scheme to every channel coefficient realization. Therefore, the ASC is chosen as the security performance metric. In contrast, the latter scenario indicates the presence of a passive eavesdropper. More specifically, Eve is a totally silent network adversary and only capable of wiretaping the Alice-Bob link. As such, Alice has no CSI knowledge of the wiretap channel, she cannot flexibly adapt her transmission rate to guarantee perfect secrecy. The SOP is correspondingly adopted as the key secrecy metric to evaluate how perfect secrecy is compromised.

Inspired by these fundamental research works, numerous research works focus on analyzing the security performance metrics over a diverse body of fading wiretap channels for the sake of better understanding the impacts of fading characteristic on secure communications, to list some, Rayleigh [9], Nakagami-*m*, Weibull [32], Rician (Nakagami-*q*) [33, 34], Hoyt (Nakagami-*n*) [35, 36], Lognormal [37], $\alpha -\mu$ (equivalently generalized Gamma or Stacy) [38–42], $\kappa -\mu$ [43–46], $\eta -\mu$ [47], generalized-${\mathcal {K}}$ (${\mathcal {K}}_G$) [48–51], extend generalized-${\mathcal {K}}$ (EGK) [52], Fisher-Snedecor ${\mathcal {F}}$ [53, 54], Gamma-Gamma [55], shadowed $\kappa -\mu$ [56], double shadowed Rician [57], Fox’s *H*-function [52], cascaded Rayleigh/Nakagami-*m*/$\alpha -\mu$ [58–60], cascaded $\kappa -\mu$ [61], $\alpha -\kappa -\mu$/$\alpha -\eta -\mu$ [62], Beaulieu-Xie [63], $\alpha -\kappa -\eta -\mu$ [64, 65], two-wave with diffuse power (TWDP) [31], *N*-wave with diffuse power (NWDP) [66], $\kappa -\mu$/Gamma [67], Fluctuating Beckmann [68], correlated Rayleigh [69], correlated composite Nakagami-*m*/Gamma [70], correlated $\alpha -\mu$ [71], correlated shadowed $\kappa -\mu$ [72], mixed $\eta -\mu$ and Málaga [73], Málaga [74–78], fluctuating two-ray (FTR) channels [79, 80]. The usage of these fading channels is examined practical and feasible in various wireless communications, such as, cellular networks [81], cellular device-to-device (D2D), vehicle-to-vehicle (V2V) communications [44], radio frequency-free space optical (RF-FSO) systems [55], mmWave communications [79], underwater acoustic communications (UAC), frequency diverse array (FDA) communications [82], body-centric fading channels, unmanned aerial vehicle (UAV) systems, land mobile satellite (LMS) [56, 83], etc.

To the authors’ best knowledge, no survey or tutorial paper has ever focused on analyzing the security performance metrics over wiretap fading channels. To this end, the main contributions of this work are listed as follows:

1. reviewing the state-of-the-art of information-theoretic security over four kinds of wiretap fading models: (i) small-scale, (ii) large-scale, (iii) composite, and (iv) cascaded.
2. displaying two clusters of security metrics to quantify information-theoretic security in the presence of active and passive eavesdropping.
3. summarizing three generic tools, i.e., the mixture Gamma (MG) distribution, the mixture of Gaussian (MoG) distribution, and Fox’s *H*-function distribution, which are used to assist the derivation of security metrics. These three tools are especially advantageous when the main channel and the wiretap channel confront different type of wiretap fading channels, e.g., the mixture of small-scale fading and composite fading models.
4. presenting the application scenarios, advantages, and limitations of the three aforementioned statistical tools. The insights drawn from the three tools demonstrate their flexibility to largely encompass the existing four kinds of wiretap fading models via adequately configuring fading channel characteristics.
5. providing four secrecy enhancement techniques, including the on-off transmission scheme, jamming approach (artificial noise (AN) and artificial fast fading (AFF)), antenna selection, and security region for Wyner’s wiretap channel model.

The remainder of this paper is organized as follows: Sect. 2 presents Wyner’s wiretap channel model, followed by Sect. 3, where the security performance metrics are presented. In Sect. 4, we review physical layer security over fading wiretap channels according to the fading channel models and also present three useful and generic tools used to assist the security metrics analysis. In Sect. 5, we introduce the secrecy enhancement schemes based on classic wiretap fading channels. Finally, Sect. 6 concludes this paper.

## Wyner’s wiretap channel model

**Fig. 1:**
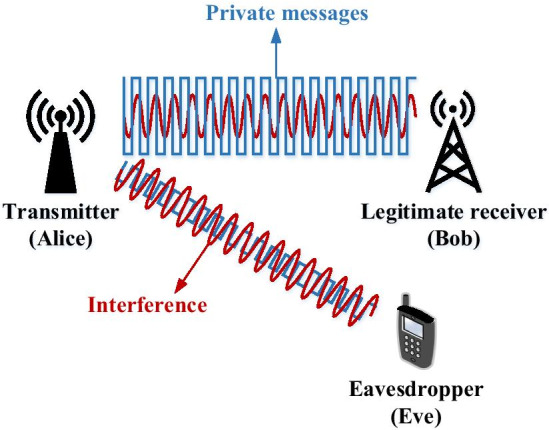
A three-node wireless wiretap system model

Consider the classic Alice-Bob-Eve wiretap channel model, as shown in Fig. 1, where Alice intends to send confidential messages to Bob in the presence of a malicious eavesdropper (Eve). The instantaneous signal-to-noise ratio (SNR) at Bob (*B*) and Eve (*E*) is expressed as $\gamma _i = {\bar{\gamma }}_i g_i, i \in \{B,E\}$, where ${\bar{\gamma }}_i$ is the average received SNR, and $g_i$ is the channel gain, which can be possibly modeled by any fading channel distributions.

## Security performance metrics

According to [9], the instantaneous secrecy capacity for one realization of the ($\gamma _B, \gamma _E$) pair over quasi-static wiretap fading channels is given by

1 $$\begin{aligned} C_s(\gamma _B,\gamma _E) = \left[ \underbrace{\log _2\left( 1+\gamma _B\right) }_{C_M}-\underbrace{\log _2\left( 1+\gamma _E\right) }_{C_W}\right] ^+, \end{aligned}$$

where $$[x]^+ \overset{\triangle }{=} \max (x,0)$$.

Based on the definition of the instantaneous secrecy capacity, security performance metrics used to evaluate the PLS over wiretap fading channels are further developed according to the availability of full CSI or partial CSI of Wyner’s wiretap model. In practice, the aforementioned two scenarios correspond to the passive eavesdropping and active eavesdropping, respectively. More specifically, it is highly questionable to have any knowledge of an evil eavesdropper’s CSI. As such, security performance metrics are classified into two categories (i) the SOP and the lower bound of SOP; and (ii) the probability of nonzero secrecy capacity (PNZ) or the intercept probability, ASC, and ergodic secrecy capacity. To this end, the two clusters of security performance metrics are vivid indicators showing whether perfect secrecy can be surely achieved or not, which are shown and compared in Table 1.

**Table 1:** PLS statistical performance metrics

Scenarios	Security metrics	CSI availability
Passive eavesdropping	SOP, lower bound of SOP	Partial CSI (only main channel)
Active eavesdropping	ASC, PNZ, intercept probability, ergodic secrecy capacity	Full CSI

### Exact security performance metrics

#### Secrecy outage probability

In the presence of a passive eavesdropper, who only listens to the main channel without sending any probing messages, Alice transmits her private messages at a constant secrecy rate $R_t$ to Bob. With this in mind, perfect secrecy can be assured only when $R_t$ falls below the instantaneous secrecy capacity $C_s$. Strikingly, the SOP is commonly seen as a key secrecy indicator used for passive eavesdropping, it measures the level that how perfect secrecy is compromised. Mathematically speaking, the SOP is the probability that the instantaneous secrecy capacity is lower than a predetermined secrecy rate $R_t$,

2 $$\begin{aligned} {\mathcal {P}}_{out}(R_t)&= Pr\left( C_s< R_t\right) \nonumber \\&= Pr(\gamma _B < 2^{R_t} \gamma _E + 2^{R_t} -1 ), \end{aligned}$$

#### The probability of nonzero secrecy capacity

The PNZ is regarded as another important secrecy metric that measures the existence of positive secrecy capacity with a probability,

3 $$\begin{aligned} {\mathcal {P}}_{nz}&= Pr \left( C_s> 0\right) \nonumber \\&= Pr(\gamma _B > \gamma _E) \nonumber \\&\mathop =^{(a)} 1- {\mathcal {P}}_{out}(R_t = 0), \end{aligned}$$

where step (*a*) is subsequently transformed from the SOP metric by setting $R_t =0$.

#### Intercept probability

In contrast to the PNZ metric, the intercept probability denotes the probability of the occurrence of an intercept event. In other words, it displays the probability of the occurrence of a negative instantaneous secrecy capacity event, which is mathematically interpreted as

4 $$\begin{aligned} {\mathcal {P}}_{int}&= Pr \left( C_s< 0\right) \nonumber \\&= Pr(\gamma _B < \gamma _E) \nonumber \\&= 1 - {\mathcal {P}}_{nz}. \end{aligned}$$

Compared to the PNZ metric, fewer works have investigated the intercept probability [84–87]. For instance, Zou and Wang in [85] studied the intercept probability of the industrial wireless sensor networks in the presence of an eavesdropping attacker.

#### Average secrecy capacity

When an active eavesdropper appears, the ASC serves as a critical measurement that guides Alice to adapt her transmission rate based on $C_M$ and $C_W$ so as to achieve perfect secrecy. In other words, the ASC is a metric that evaluates how much achievable secrecy rate can be guaranteed. It is mathematically defined as

5 $$\begin{aligned} {\bar{\mathcal {C}}} = {\mathcal {E}}[C_s(\gamma _B,\gamma _E)], \end{aligned}$$

where ${\mathcal {E}}[\cdot ]$ is the expectation operator.

### Security performance bounds

The usage of non-elementary functions is widely used to describe the statistical characteristics of fading models, e.g., the $\kappa -\mu$ distribution with the modified Bessel function of the first kind in its probability density function (PDF) and the generalized Marcum *Q* function in its cumulative distribution function (CDF), and the EGK distribution with the extended incomplete Gamma function in its PDF. Obviously, the existence of those special functions makes it highly intractable to deduce the security performance metrics embedded with both the PDF and CDF of the instantaneous SNR $\gamma _i$ simultaneously. As a result, the acquisition of exact security performance metrics with closed-form expressions is a challenging issue, security performance bounds, including the lower bound of the SOP and ergodic secrecy capacity, are in turn adopted as effective alternatives in many works.

#### The lower bound of SOP

The exact SOP can be accurately approximated by its lower bound when (i) the given transmission rate tends to zero, i.e., $R_t \rightarrow 0$; and (ii) Eve is closely located to Alice, which can be physically interpreted as Eve having an extremely high average received SNR, i.e., ${\bar{\gamma }}_E \rightarrow \infty$. In this context, the lower bound of SOP can be computed as

6 $$\begin{aligned} {\mathcal {P}}_{out}^L&= Pr(\gamma _B< 2^{R_t} \gamma _E ) \nonumber \\&< Pr(\gamma _B < 2^{R_t} \gamma _E + 2^{R_t} -1 ). \end{aligned}$$

Such an alternative has been widely investigated (see references [38, 39, 48, 53, 60]), and was shown to provide a fairly tight approximation.

#### Ergodic secrecy capacity

As an appropriate secrecy measure to characterize the time-varying feature of wireless channels, the ergodic secrecy capacity is consequently utilized to quantify the ergodic features of wireless channels [42, 88–91]. The ergodic secrecy capacity is mathematically evaluated by averaging the channel capacity over all fading channel realizations, which is mathematically computed as follows,

7 $$\begin{aligned} {\mathcal {E}}(C_s) = \left[ {\mathcal {E}}[\log _2(1+\gamma _B)] - {\mathcal {E}}[\log _2(1+\gamma _E)\right] ^+. \end{aligned}$$

For instance, the authors in [92] investigated the ergodic secrecy rate of downlink multiple-input multiple-output (MIMO) systems with limited CSI feedback. Similarly, considering the zero-forcing (ZF) beamforming at Alice and ZF detectors at Bob and Eve, the upper and lower bounds of the ergodic secrecy capacity of MIMO systems were explored in [90].

## Secrecy characterization

In wireless communication systems, the transmitted signals are reflected, diffracted, and scattered from objects that are present on their path to the receivers. The received signals experience fading (multipath) and shadowing (signal power attenuation or pathloss) phenomena, which pose destructive and harmful impacts at the receiver sides. The essence of PLS lies in reversely using the impairments of wireless channels as secrecy enhancement means.

Under the assumption that the main and wiretap channels undergo independent fading conditions, this section mainly presents the security performance analysis over wiretap fading channels according to the following four categories.

### Exact secrecy analysis

#### Small-scale fading channels

The random changes in signal amplitude and phase from the spatial positioning between a receiver and a transmitter is referred to small-scale fading. The well-known small-scale fading models are Rayleigh, Nakagami-*m*, Rician, $\alpha -\mu$, etc. The simple and tractable form of these models makes small-scale fading appealing and popular in the security and reliability performance analysis. Examples can be found in [9, 33, 38–41], where the SOP, PNZ, and ASC metrics are analyzed with either closed-form or highly tight approximated expressions. It is noteworthy of mentioning that the $\alpha -\mu$ distribution can be reduced to Rayleigh ($\alpha =2,\mu =1$), Nakagami-*m* ($\alpha =2,\mu =m$), Weibull ($\alpha$ is the fading parameter, $\mu =1$), and Gamma ($\alpha =1$, $\mu$ is the fading parameter) distributions by properly attributing the values of $\alpha$ and $\mu$. To this end, the applicability and flexibility of the $\alpha -\mu$ distribution have been well explored in the literature. Besides, the TWDP fading model is also of high flexibility as it includes Rayleigh, Rician, and hyper-Rayleigh as special cases. The TWDP model characterizes propagating scenarios where the received signal contains two strong, specular multipath waves, moreover, it can also model a link worse than Rayleigh fading. More importantly, it provides a good fit to the the real-world frequency-selective fading data from wireless sensor networks [93]. The PLS investigation over TWDP wiretap fading channels was studied in [31]. Apart from the aforementioned works, in [94], the authors studied the effect of eavesdroppers’ location uncertainty on the SOP metric, where Eve is located in a ring-shaped area around Alice and undergoes Rayleigh fading.

Another interesting direction of PLS over small-scale fading channels lies in the secrecy investigation over correlated fading channels. The correlation is caused due to the distances between Bob and Eve, or the scattering environments. The physical correlation essentially makes the fading statistics, i.e., the mathematical representation of the joint PDF of $\gamma _B$ and $\gamma _E$, fairly complex and eventually makes it intractable and highly difficult to obtain exact closed-form security performance metrics, instead, secrecy performance bounds are derived (see references [69, 71]).

#### Large-scale fading channels

The so-called large-scale fading results from signal attenuation due to signal propagation over large distance and diffraction around large objects, e.g., hills, mountains, forests, billboards, buildings, etc., in the propagation path. One widely studied example of large-scale fading channels is the Lognormal distribution. However, its complex mathematical form hinders the derivation of exact reliability and security performance expressions. For instance, Pan et al. [37] investigated the PLS over non-small scale fading channels, wherein independent/correlated Lognormal fading channels and composite fading channels were considered and approximated security performance representations were derived.

#### Composite fading channels

Different from the small-scale (fading) and large-scale (shadowing) fading models, composite fading models are proposed to account for the effects of both small-scale and large-scale fading simultaneously. For instance, Kumar et al. in [44] presented the SOP, PNZ, and ASC over $\kappa -\mu$ fading channels and explored the obtained results in several wireless communication scenarios, including cellular D2D, body area networks (BAN), and V2V. Moualeu and Hamouda in [46] subsequently extended the results in [44] to the single-input multiple-output (SIMO) scenario and derived the ASC and lower bound of SOP. More recently, to elaborate the shadowing effect of wireless channels, the authors in [57, 72] investigated the security performance over the shadowed Rician and $\kappa -\mu$ wiretap fading channels.

Other widely used fading models, e.g., generalized-${\mathcal {K}}$, Rayleigh/Lognormal (RL), Nakagami-*m*/Lognormal (NL), Gamma-Gamma, and Fisher-Snedecor ${\mathcal {F}}$, are examined in practice to model the channel-induced physical layer dynamics. For example, the Fisher-Snedecor ${\mathcal {F}}$ fading model was proposed in [95] to characterize D2D communications, where its simplicity and feasibility are compared with the generalized-${\mathcal {K}}$ fading model. Similarly, the Gamma-Gamma, mixed $\eta -\mu$ and Málaga, and Málaga distributions were shown feasible to accurately model the RF-FSO links, and the security performance analysis of RF-FSO systems over these fading models are explored in [73–76, 96]. To encompass more special models in one distribution, one can find that [62, 64, 65], respectively, analyzed the security performance metrics over $\alpha -\eta -\mu$, $\alpha -\kappa -\mu$, and $\alpha -\eta -\kappa -\mu$ fading models. For instance, the $\alpha -\eta -\kappa -\mu$ model can be reduced to the Rayleigh, Nakagami-*m*, Rician, $\kappa -\mu$, $\eta -\mu$, $\alpha -\mu$, etc. Those models are highly valuable and flexible. However, its complex mathematical representation of characteristics makes it difficult to derive the exact closed-form security metrics.

#### Cascaded fading channels

Cascaded fading models were found feasible to characterize the multi-hop non-regenerative amplify-and-forward (AF) relaying with fixed gain, the propagation in the presence of keyholes, the keyhole/pinhole phenomena in MIMO systems, and the reconfigurable intelligent surface (RIS)-aided wireless systems [97–99]. Yang et al. [97] modeled the RIS-aided main link as a multiplication of two Rayleigh distributed random variables. For vehicular networks, Ai et al. [58] considered the double Rayleigh fading channels and analyzed the ASC metric. Regarding other works over cascaded Nakagami-*m*, cascaded Fisher-Snedecor ${\mathcal {F}}$, and cascaded $\alpha -\mu$ wiretap fading channels, readers can refer to [58–61, 87, 100]. As discussed earlier, the cascaded $\alpha -\mu$ fading channel similarly includes the cascaded Rayleigh, cascaded Nakagami-*m*, cascaded Weibull, and cascaded Gamma distributions. The authors of [60] studied the SOP, PNZ, and ASC performances with closed-form expressions, which are given in terms of Fox’s *H*-function. The obtained results therein are identical to the exact analytical representations given in [87, 100]. In [61], Tashman et al. considered multiple eavesdroppers and investigated the SOP and PNZ metrics with closed-form expressions over cascaded $\kappa -\mu$ wiretap fading channels.

As shown in Table 2, the existing research works focusing on analyzing security performance metrics over wiretap fading channels are summarized and their contributions are highlighted.

**Table 2:** Major information-theoretic secrecy analysis works over the classic wiretap fading channels

Year	References	Contributions
2008	Bloch et al. [9]	Derived simple and exact SOP, PNZ, and ASC closed-form expressions over **Rayleigh** fading channels
2013	Liu [32, 33]	Derived the PNZ over **Rician** and **Weibull** fading channels
2014	Wang et al. [31]	Derived the ASC and SOP over **TWDP** fading channels
2015–2018	Lei et al. [38, 40], Kong et al. [39, 41]	Analyzed the SOP, lower bound of SOP, PNZ, and ASC over $\alpha -\mu$ fading channels
2016	Pan et al. [37]	Proposed an highly accurate approximated secrecy solution over **lognormal** fading channels
	Bhargav et al. [44]	Derived the lower bound of SOP and PNZ over $\kappa -\mu$ fading channels
	Lei et al. [48–50]	Analyzed the security metrics over **generalized**-${\mathcal {K}}$ fading channels
2017	Saber and Sadough [74]	Derived the SOP, PNZ, and ASC over the **Málaga** fading channels
2018	Kong and Kaddoum [53]	Derived the SOP, lower bound of SOP, PNZ and ASC over **Fisher-Snedecor** ${\mathcal {F}}$ fading channels
	Kong et al. [60]	Derived closed-form expressions for the SOP, PNZ, and ASC over **cascaded** $\alpha -\mu$ fading channels
	Mathur et al. [65]	Derived the ASC and SOP over $\alpha -\eta -\kappa -\mu$ fading channels
2019	Kong & Kaddoum [36]	Analyzed the security metrics with the assistance of the **MG** distribution
	Kong et al. [52]	Analyzed the security metrics over a general and flexible **Fox’s** *H*-**function** fading channels
	Moualeu et al. [62]	Derived closed-form expressions of lower bound of SOP and their asymptotic behavior over the $\alpha -\eta -\mu$ & $\alpha -\kappa -\mu$ fading channels
	Zeng et al. [79], Zhao et al. [80]	Analyzed the security metrics over the **FTR** fading channels
2020	Kong et al. [101]	Proposed a unified secrecy analysis framework with the help of **MoG** distribution
	Sánchez et al. [56]	Derived the closed-form expressions of SOP and ASC metrics over **shadowed** $\kappa -\mu$ fading channels
	Sánchez et al. [66]	Derived the exact and asymptotic SOP behavior over **NWDP** fading channels
	Tashman et al. [61]	Derived the SOP and PNZ over cascaded $\kappa -\mu$ fading channels
	Badarneh et al. [54]	Derived the ASC, PNZ, and SOP over **Nakagami**-*m*/**Fisher Snedecor** ${\mathcal {F}}$, **Fisher Snedecor** ${\mathcal {F}}$/**Nakagami**-*m*, and **Nakagami**-*m*/**Nakagami**-*m* fading channels
2021	Ai et al. [78]	Derived the SOP and PNZ over correlated **Málaga** fading channels

The bold items are used to highlight the contributions of the cited works

### Generic secrecy analysis tools

With the above in mind and under the assumption that the main and wiretap channels undergo independent fading conditions, this subsection will present three useful and flexible distributions, which can largely encompass the aforementioned fading channel models by properly attributing their parameters. It is proved in literature that they are general and advantageous to assist the theoretical analysis of security metrics.

#### Mixture Gamma (MG) distribution

According to [102, 103], the instantaneous received SNR $\gamma$ over wireless Rayleigh, Nakagami-*m*, NL, $\kappa -\mu$, Hoyt, $\eta -\mu$, Rician, ${\mathcal {K}}$, ${\mathcal {K}}_G$, $\kappa -\mu$/Gamma, $\eta -\mu$/Gamma, and $\alpha -\mu$/Gamma fading channels can be reformulated using the MG distribution, whereas the PDF and CDF of the instantaneous received SNR $\gamma$ are denoted as $f(\gamma )$ and $F(\gamma )$ and given by

8 $$\begin{aligned} f(\gamma )&= \sum \limits _{l=1}^{L} \alpha _{l}\gamma ^{\beta _{l} -1}\exp (-\zeta _{l}\gamma ), \end{aligned}$$

9 $$\begin{aligned} F(\gamma )&= \sum \limits _{l=1}^{L} \alpha _{l} \zeta _{l}^{-\beta _{l}} \Upsilon (\beta _{l},\zeta _{l}\gamma ), \end{aligned}$$

where *L* is the number of terms in the mixture, while $\alpha _{l}, \beta _{l}$, and $\zeta _{l}$ are the parameters of the *l*th Gamma component. $\Upsilon (\cdot ,\cdot )$ is the lower incomplete Gamma function.

Lei et al. [49] used the MG distribution to assist the information-theoretic security performance analysis over wiretap generalized-${\mathcal {K}}$ fading channels. Motivated by [36], the security metrics over the FTR and Málaga turbulence fading channels [74, 79] can be similarly derived using the MG distribution.

#### Mixture of Gaussian (MoG) distribution

Based on the unsupervised expectation-maximization (EM) learning algorithm, the MoG distribution is essentially beneficial when the characteristics of fading channels are unavailable. In [104], the authors modeled the RL, NL, $\eta -\mu$, $\kappa -\mu$, and shadowed $\kappa -\mu$ fading channels using the MoG distribution. The findings of [104] showcase that the MoG distribution is, especially advantageous to approximate any arbitrarily shaped non-Gaussian density and can accurately model both composite and non-composite channels in a simple expression.

Assuming the instantaneous SNR $\gamma$ follows the MoG distribution, its PDF and CDF are given by

10 $$\begin{aligned} f(\gamma )&= \sum \limits _{l=1}^{C} \frac{w_l}{\sqrt{8\pi {\bar{\gamma }}}\eta _l \sqrt{\gamma } }\exp \left( -\frac{(\sqrt{\gamma /{\bar{\gamma }}} - \mu _l)^2}{2\eta _l^2}\right) , \end{aligned}$$

11 $$\begin{aligned} F(\gamma )&= \sum \limits _{l=1}^{C}w_l \Phi \left( \frac{\sqrt{ \gamma /{\bar{\gamma }}} - \mu _l}{\eta _l} \right) , \end{aligned}$$

where *C* represents the number of Gaussian components. $w_l>0$, $\mu _l$, and $\eta _l$ are the *l*th mixture component’s weight, mean, and variance with $\sum _l^C w_l = 1$, $\Phi (x)$ is the CDF of the standard normal distribution.

#### Fox’s *H*-function distribution

For known fading characteristics, the Fox’s *H*-function distribution is a general and flexible tool. It is reported in [52, 105–107] that many well-known distributions in the literature, e.g., Rayleigh, Exponential, Nakagami-*m*, Weibull, $\alpha -\mu$, Gamma, Fisher-Snedecor ${\mathcal {F}}$, Chi-square, cascaded Rayleigh/Nakagami-*m*/$\alpha -\mu$, Gamma-Gamma, Málaga, ${\mathcal {K}}_G$, EGK, etc., can be represented using Fox’s *H*-function distribution. Interested readers are suggested to refer to Table 3.

**Table 3:** Fox’s *H*-equivalents of typical and generalized statistical models for instantaneous received SNR $\gamma _i, i \in \{B,E \}$, and ${\bar{\gamma }}_i$ is the average SNR

Model	${\boldsymbol{{\mathcal {K}}}}$	$\boldsymbol{\lambda}$	***m*** ***n***	${\boldsymbol{\mathfrak {a}}}$	${\boldsymbol{\mathfrak {b}}}$
			***p*** ***q***	${\boldsymbol{\mathscr {A}}}$	${\boldsymbol{\mathscr {B}}}$
Rayleigh	$\frac{1}{{\bar{\gamma }}_i}$	$\frac{1}{{\bar{\gamma }}_i}$	1 0	–	0
			0 1	–	1
Nakagami	$\frac{m}{\Gamma (m){\bar{\gamma }}_i}$	$\frac{m}{{\bar{\gamma }}_i}$	1 0	–	$m -1$
			0 1	–	1
Weibull	$\frac{\Gamma (1+\frac{2}{\alpha })}{{\bar{\gamma }}_i}$	$\frac{\Gamma (1+\frac{2}{\alpha })}{{\bar{\gamma }}_i}$	1 0	–	$1 - \frac{2}{\alpha }$
			0 1	–	$\frac{2}{\alpha }$
$\alpha$-$\mu$	$\frac{\Gamma (\mu + \frac{2}{\alpha })}{\Gamma (\mu )^2{\bar{\gamma }}_i}$	$\frac{\Gamma (\mu +\frac{2}{\alpha })}{\Gamma (\mu ){\bar{\gamma }}_i}$	1 0	–	$\mu - \frac{2}{\alpha }$
			0 1	–	$\frac{2}{\alpha }$
Maxswell	$\frac{3}{\sqrt{\pi }{\bar{\gamma }}_i}$	$\frac{3}{2{\bar{\gamma }}_i}$	1 0	–	$\frac{1}{2}$
			0 1	–	1
$N*$($\alpha$-$\mu )$	$\prod \limits _{i=1}^N\frac{\Gamma (\mu _i + \frac{2}{\alpha _i})}{\Gamma (\mu _i)^2{\bar{\gamma }}_i}$	$\prod \limits _{i=1}^N\frac{\Gamma (\mu _i + \frac{2}{\alpha _i})}{\Gamma (\mu _i){\bar{\gamma }}_i}$	*N* 0	–	$(\mu _1 - \frac{2}{\alpha _1},\cdots ,\mu _N - \frac{2}{\alpha _N})$
			0 *N*	–	$(\frac{2}{\alpha _1},\cdots , \frac{2}{\alpha _N})$
Fisher-Snedecor ${\mathcal {F}}$	$\frac{m}{m_s{\bar{\gamma }}_i \Gamma (m)\Gamma (m_s)}$	$\frac{m}{m_s{\bar{\gamma }}_i }$	1 1	$-m_{s}$	1
			1 1	$m-1$	1
Generalized-${\mathcal {K}}$	$\frac{m_l m_{sl}}{\Gamma (m_l)\Gamma (m_{sl}){\bar{\gamma }}_i}$	$\frac{m_1m_2}{{\bar{\gamma }}_i}$	2 0	–	$(m_l-1 ,m_{s1} - 1)$
			0 2	–	(1, 1)
EGK	$\frac{\Gamma (m + \frac{1}{\xi }) \Gamma (m_s + \frac{1}{\xi _s})}{{\bar{\gamma }}_i\Gamma (m)^2\Gamma (m_s)^2}$	$\frac{\Gamma (m + \frac{1}{\xi }) \Gamma (m_s + \frac{1}{\xi _s})}{{\bar{\gamma }}_i\Gamma (m)\Gamma (m_s)}$	2 0	–	$(m - \frac{1}{\xi }, m_{s} - \frac{1}{\xi _{s}})$
			0 2	–	$(\frac{1}{\xi }, \frac{1}{\xi _{s}})$

Assuming $\gamma$ follows Fox’s *H*-function distribution, its PDF and CDF are given by

12 $$\begin{aligned} f(\gamma )&= {\mathcal {K}} H_{p,q}^{m,n} \left[ {{\mathcal {C}} \gamma \left| {\begin{array}{*{20}c} {(a_\tau +A_\tau ,A_\tau )_{\tau = 1:p}} \\ {(b_\varsigma +B_\varsigma ,B_\varsigma )_{\varsigma =1:q}} \\ \end{array}} \right. } \right] , \end{aligned}$$

13 $$\begin{aligned} F(\gamma )&= \frac{{\mathcal {K}}}{{\mathcal {C}}} H_{p+1,q+1}^{m,n+1} \left[ {{\mathcal {C}} \gamma \left| {\begin{array}{*{20}c} {(1,1),(a_\tau +A_\tau ,A_\tau )_{\tau = 1:p}} \\ {(b_\varsigma +B_\varsigma ,B_\varsigma )_{\varsigma =1:q},(0,1)} \\ \end{array}} \right. } \right] , \end{aligned}$$

where $H_{p,q}^{m,n}[.]$ is the univariate Fox’s *H*-function [108, Eq. (8.4.3.1)], ${\mathcal {K}} > 0$ and ${\mathcal {C}}$ are constants such that $\int _0^\infty f (\gamma ) d \gamma = 1$. $A_i > 0$ for $i=1,\cdots , p$, $B_l > 0$ for $l = 1, \cdots , q$, $0 \le m \le q$, and $0 \le n \le p$. For notational convenience, let ${\mathfrak {a}} = (a_1,\cdots ,a_p)$, ${\mathscr {A}}= (A_1,\cdots ,A_p)$, ${\mathfrak {b}} = (b_1,\cdots ,b_q)$, and ${\mathscr {B}} = (B_1,\cdots ,B_q)$. Thus, hereafter the Fox’s *H*-function is denoted as ${\mathcal {H}}_{p,q}^{m,n}({\mathcal {K}}, {\mathcal {C}}, {\mathfrak {a}}, {\mathscr {A}}, {\mathfrak {b}}, {\mathscr {B}})$.

**Fig. 2:**
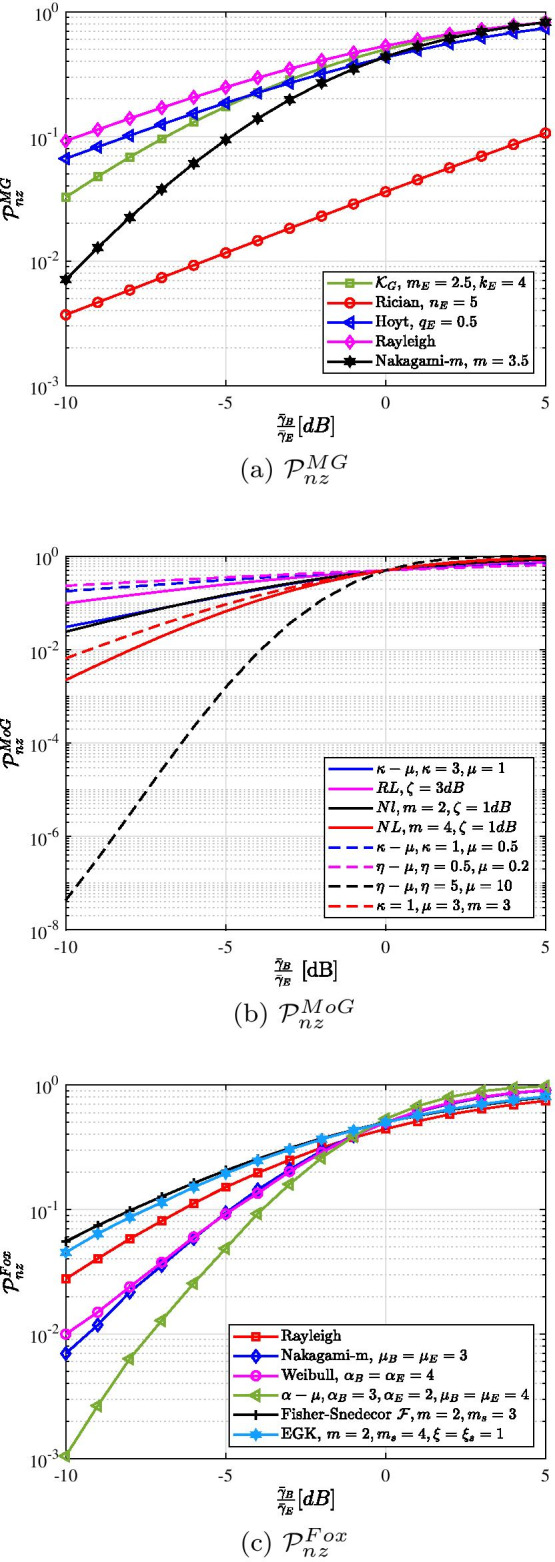
Illustration of ${\mathcal {P}}_{nz}$ versus $\frac{{\bar{\gamma }}_B}{{\bar{\gamma }}_E}$ using the MG, MoG, and Fox’s *H*-function distributions when ${\bar{\gamma }}_E$ = 0 dB, **a** the main channel undergoes ${\mathcal {K}}_G$ ($m_B = 2.5, k_B = 4$) fading while the wiretap channel experiences ${\mathcal {K}}_G$, Rician, Hoyt, Rayleigh, and Nakagami-*m* ($m = 3.5$) fading; **b** the main and wiretap channels undergo same fading while using the MoG distribution; and **c** the main and wiretap channels undergo same fading while using the Fox’s *H*-function distribution

To compare the security performance analysis using the three aforementioned approaches, the PNZ metric is taken as an example. Provided that the main and wiretap links undergo the same fading conditions, the PNZ expressions are derived in terms of the Gauss Hypergeometric function [36, Eq. (7)], error function [101, Eq. (9)], and Fox’s *H*-function [52, Eq. (16)]. In Fig. 2, we plotted the PNZ performance versus ${\bar{\gamma }}_B$ for different fading channel models. Their tightness and accuracy have already been individually presented and confirmed in [36, 52, 101].

##### *Remark*

Conclusively speaking, the MG, MoG, and Fox’s *H*-function distributions have demonstrated their feasibility and applicability when analyzing security performance metrics. They all are valid when the main channel and wiretap channel are subjected to different wireless fading channels. Their advantages and limitations are listed in Table 4.

**Table 4:** Comparisons among the MG, MoG, and Fox’s *H*-function distributions

	Applicable scenarios	Advantages	Limitations
MG	Exactly known fading models	Highly accurate solutions with simple expressions	Accuracy depends on *L*
MoG	Unavailability of fading model	Highly accurate approximated solution	Accuracy relies on *C*
Fox	Exactly known and transformable models	Exact and general solution	Inflexibility to some composite fading channels

Note that the three aforesaid solutions are unfeasible when the main and wiretap channels are correlated.

### Outdated and imperfect and correlated CSI

The aforementioned works mainly focus on the scenario that perfect CSI is available at all parties. Such an assumption is unrealistic in practice, since outdated CSI and imperfect CSI are the general cases due to the time varying nature of wireless channels and channel estimation errors.

In [109], the effects of outdated CSI on security performance were investigated over multiple-input single-output (MISO) systems when the transmit antenna selection (TAS) scheme is applied at Alice. The obtained analytical results show that the diversity gain of using multiple antenna techniques cannot be achieved when the CSI is outdated during the TAS process. Later on in [110], Hu et al. adopted the on-off-based transmission scheme at Alice to efficiently take advantage of the useful information in the outdated CSI. Alice does transmission only when she has a better link to Bob compared with that to Eve. Perfect knowledge of the main and wiretap channel CSI are always favorable, but the existence of noise in the channel estimation process makes it an unrealistic assumption. The impacts of imperfect CSI have been widely explored in diverse research topics, e.g., imperfect CSI in the AN-assisted training and communications [111], imperfect CSI with an active full-duplex eavesdropper [112], imperfect CSI in a mixed RF/FSO system [55], etc.

Apart from the above two scenarios, the correlation between the main channel and wiretap channel also attracts a growing body of research interests. Channel correlation at the physical layer is often observed, which is mainly caused by the antenna deployments (e.g., insufficient antenna spacing in small mobile units equipped with space and polarization antenna diversity), proximity of the legitimate and illegitimate receivers, and random scatters around them [69]. The correlation is mathematically modeled with the correlated wiretap fading channel models. For example, Jeon et al. in [69] used the correlated Rayleigh fading wiretap channel and explored the secrecy capacity bounds. The results quantitatively showcased how much of secrecy capacity is lost due to channel correlation. In continuation of this work, the security performance analysis over correlated Nakagami-*m*, correlated $\alpha -\mu$, correlated shadowed $\kappa -\mu$, and correlated Málaga fading channels are explored in [71, 72, 78, 113].

## Secrecy enhancement approaches

The essence of PLS is to utilize the impairments (e.g., fading, noise, interference, and path diversity) of wireless channels to enhance security. In this section, we mainly focus on comparing the existing secrecy enhancement techniques suitable for wiretap channels.

### On-off transmission scheme

Consider the imperfect channel estimation, He and Zhou in [89] first proposed the on-off transmission scheme to improve the reliability and security performance. The principle of on-off transmission lies in the comparison between the estimated instantaneous SNRs at Bob and Eve, i.e., ${\hat{\gamma }}_B$ and ${\hat{\gamma }}_E$, and two given corresponding thresholds i.e., $\mu _B$ and $\mu _E$. More specifically, only when the condition ${\hat{\gamma }}_B \ge \mu _B$ and ${\hat{\gamma }}_E \le \mu _E$ meet, the ‘on’ mode at Alice is then activated, otherwise, Alice is in ‘off’ mode. The on-off transmission scheme is an appealing enabler to allow the SOP metric to be arbitrarily small. Building on He’s work, the on-off transmission is thereafter widely investigated in the following works [110, 114–116], where the imperfect CSI, outdated CSI, and correlated CSI are considered.

### Jamming approach

Assuming the transmitter has more antennas than the eavesdropper, Goel and Negi proposed the concept of artificial noise (AN) [117]. The principle of AN lies in that the transmitter allocates some of its available power to generate AN to confuse passive eavesdroppers. Similarly, Wang et al. in [118] proposed the artificial fast fading (AFF) secrecy enhancement scheme, where the randomized beamforming is employed at the transmitter to ‘upgrade’ the main channel to an AWGN one and degrade the wiretap channel to a fast fading channel.

Unlike the aforesaid transmitting beamforming-based techniques, i.e., AN and AFF, the quality of the wiretap link is further degraded by allocating part of the transmitting resources (i.e., power or antennas) at the transmitter, specifically to Eve. Based on the survey papers [3, 19], one can conclude that jamming is a useful means to enhance the PLS. Considering the three-node wiretap fading channel, jamming can be alternatively realized by a full-duplex Bob, where Bob would receive signals from Alice and send jamming signals (e.g., noise) to Eve in order to reduce Eve’s received SNR’s quality [119]. Bob and Eve usually only act purely as a legitimate receiver or an illegitimate evil eavesdropper. However, in practice, they might behave with multiple roles. For instance, in [112], an active eavesdropper operates in full-duplex mode so that it can send jamming signals to degrade the legitimate receiver’s SNR, while in [120, 121], an untrustworthy relay works as a relay and eavesdropper simultaneously in a bidirectional cooperative network.

### Antenna selection technique

In multiple-antenna systems, TAS is seen as an effective way for reducing hardware complexity while boosting diversity benefits. In [113, 116, 122–127], TAS is deployed as a secrecy enhancement solution in MIMO systems. There exist three kinds of TAS schemes, i.e., (i) the antenna that maximizes the instantaneous output SNR at Bob is selected (see [122, 123]); (ii) more than one single antenna are selected (see [124]); and (iii) a general order of antenna is selected (see [126]).

Unlike the works [122–125] assuming that the multi-antenna channels are independent, quite recently, Si et al. consider antenna correlation in [116], where the exact and asymptotic SOP are derived with consideration of three diversity combining schemes, namely maximal ratio combining (MRC), selection combining (SC), and equal gain combining (EGC) at Bob. This work is extended in [113], where the authors continuously consider the joint antenna and channel correlation, while the relationship between the correlation and the SOP is analytically established.

### Protected zones

Protected zones (equivalently, secrecy region) mean a geometrical region (see [91, 128]), defined as the legitimate receiver’s locations having a certain guaranteed level of secrecy, or an area where the set of ordered nodes can safely communicate with typical destination, for a given secrecy outage constraint [42, 129].

## Concluding remarks

In this paper, we have comprehensively reviewed the development of PLS over various wiretap fading channels. Based on the characteristics of wireless channels, research works focusing on investigating security performance metrics are thereafter classified into four categories: (i) small-scale fading; (ii) large-scale fading; (iii) cascaded fading; and (iv) composite fading models. After comparing some significant existing and ongoing research works, we introduced three valuable and practical approaches, i.e., the MG, MoG, and Fox’s *H*-function distributions, to simplify the analysis of security performance metrics. The three approaches are highly beneficial and advantageous since they can broadly encompass the existing fading models. Besides, we discussed four secrecy enhancement techniques deployed on Wyner’s wiretap channel model, including on-off transmission, jamming approach, TAS technique, and protected zones. Hopefully, this paper can serve as a valuable reference for interested readers on better understanding the physical layer security over wiretap fading channels.

## Abbreviations

AN: Artificial noise
AF: Amplify-and-forward
AFF: Artificial fast fading
ASC: Average secrecy capacity
D2D: Device-to-device
EGK: Extended generalized ${\mathcal {K}}$
MG: Mixture of Gamma
MoG: Mixture of Gaussian
SNR: Signal-to-noise ratio
MIMO: Multiple-input multiple-output
MISO: Multiple-input single-output
CDF: Cumulative distribution function
PDF: Probability density function
TAS: Transmit antenna selection
SC: Selection combining
MRC: Maximal ratio combining
EGC: Equal gain combining
SOP: Secrecy outage probability
PNZ: Probability of nonzero secrecy capacity
AWGN: Additive white Gaussian noise
ITU: International Telecommunications Union
CSI: Channel state information
RF-FSO: Radio frequency-free space optical
OSI: Open systems interconnection
RFID: Radio-frequency identification
IRS: Intelligent reconfigurable surfaces
TDMA: Time-division multiple-access
TWDP: Two-wave with diffuse power
NWDP: *N*-wave with diffuse power
V2V: Vehicle-to-vehicle
FTR: Fluctuating two-ray
UAC: Underwater acoustic communications
UAV: Unmanned aerial vehicle
LMS: Land mobile satellite
PLS: Physical layer security

## References

[1] ITU, Digital trends in Europe 2021ICT trends and developments in Europe, 2017–2020. Technical report. https://www.itu.int/en/myitu/Publications/2021/02/05/14/28/Digital-trends-in-Europe-2021

[2] Zou Y, Zhu J, Wang X, Hanzo L (2016). A survey on wireless security: technical challenges, recent advances, and future trends. Proc. IEEE.

[3] Huo Y, Tian Y, Ma L, Cheng X, Jing T (2018). Jamming strategies for physical layer security. IEEE Wirel. Commun..

[4] Liu R, Trappe W (2010). Securing Wireless Communications at the Physical Layer.

[5] Zhou X, Song L, Zhang Y (2013). Physical Layer Security in Wireless Communications.

[6] Shannon CE (1949). Communication theory of secrecy systems. Bell Syst. Tech. J..

[7] Wyner AD (1975). The wire-tap channel. Bell Syst. Tech. J..

[8] Leung-Yan-Cheong S, Hellman ME (1978). The Gaussian wire-tap channel. IEEE Trans. Inf. Theory.

[9] Bloch M, Barros J, Rodrigues MRD, McLaughlin SW (2008). Wireless information-theoretic security. IEEE Trans. Inf. Theory.

[10] Gopala PK, Lai L, El Gamal H (2008). On the secrecy capacity of fading channels. IEEE Trans. Inf. Theory.

[11] Bassily R, Ekrem E, He X, Tekin E, Xie J, Bloch MR, Ulukus S, Yener A (2013). Cooperative security at the physical layer: a summary of recent advances. IEEE Signal Process. Mag..

[12] P. Mukherjee, R. Tandon, S. Ulukus, in *Physical-Layer Security with Delayed, Hybrid, and Alternating Channel State Knowledge*. ed. by R.F. Schaefer, H. Boche, A. Khisti, H.V. Poor (Cambridge University Press, Cambridge, 2017), pp. 200–230

[13] Poor HV, Schaefer RF (2017). Wireless physical layer security. Proc. Natl. Acad. Sci..

[14] Bloch M, Barros J (2011). Physical-layer Security: From Information Theory to Security Engineering.

[15] He B, Zhou X, Abhayapala TD (2013). Wireless physical layer security with imperfect channel state information: a survey. ZTE Commun..

[16] Shiu Y, Chang SY, Wu H, Huang SC, Chen H (2011). Physical layer security in wireless networks: a tutorial. IEEE Wirel. Commun..

[17] D.P.M. Osorio, J.D.V. Sánchez, H. Alves, Physical-layer security for 5G and beyond, in *Wiley 5G Ref*, pp. 1–19 (2019)

[18] Mukherjee A, Fakoorian SAA, Huang J, Swindlehurst AL (2014). Principles of physical layer security in multiuser wireless networks: a survey. IEEE Commun. Surv. Tutor..

[19] M. Atallah, G. Kaddoum, L. Kong, A survey on cooperative jamming applied to physical layer security, in *IEEE ICUWB, Montreal, Quebec, Canada*, pp. 1–5 (2015)

[20] Chen X, Zhong C, Yuen C, Chen H (2015). Multi-antenna relay aided wireless physical layer security. IEEE Commun. Mag..

[21] Liu Y, Chen H, Wang L (2017). Physical layer security for next generation wireless networks: theories, technologies, and challenges. IEEE Commun. Surv. Tutor..

[22] Schaefer RF, Boche H, Poor HV (2015). Secure communication under channel uncertainty and adversarial attacks. Proc. IEEE.

[23] Hyadi A, Rezki Z, Alouini M (2016). An overview of physical layer security in wireless communication systems with CSIT uncertainty. IEEE Access.

[24] Chen X, Ng DWK, Gerstacker WH, Chen H (2017). A survey on multiple-antenna techniques for physical layer security. IEEE Commun. Surv. Tutor..

[25] Wu Y, Khisti A, Xiao C, Caire G, Wong K, Gao X (2018). A survey of physical layer security techniques for 5G wireless networks and challenges ahead. IEEE J. Sel. Areas Commun..

[26] Nguyen BV, Jung H, Kim K (2018). Physical layer security schemes for full-duplex cooperative systems: State of the art and beyond. IEEE Commun. Mag..

[27] Chen R, Li C, Yan S, Malaney R, Yuan J (2019). Physical layer security for ultra-reliable and low-latency communications. IEEE Wirel. Commun..

[28] Dai B, Li C, Liang Y, Poor HV, Shamai S (2020). Enhancing physical layer security via channel feedback: a survey. EURASIP J. Wirel. Commun. Netw..

[29] Li B, Fei Z, Zhou C, Zhang Y (2020). Physical-layer security in space information networks: a survey. IEEE Internet Things J..

[30] Bloch M, Günlü O, Yener A, Oggier F, Poor HV, Sankar L, Schaefer RF (2021). An overview of information-theoretic security and privacy: metrics, limits and applications. IEEE J. Sel. Areas Inf. Theory.

[31] Wang L, Yang N, Elkashlan M, Yeoh PL, Yuan J (2014). Physical layer security of maximal ratio combining in two-wave with diffuse power fading channels. IEEE Trans. Inf. Forensics Secur..

[32] X. Liu, Probability of strictly positive secrecy capacity of the Weibull fading channel, in *IEEE GLOBECOM, Atlanta, GA, USA*, pp. 659–664 (2013)

[33] Liu X (2013). Probability of strictly positive secrecy capacity of the Rician-Rician fading channel. IEEE Wirel. Commun. Lett..

[34] S. Iwata, T. Ohtsuki, P. Kam, Performance analysis of physical layer security over Rician/Nakagami-*m* fading channels, in *2017 IEEE 85th Vehicular Technology Conference (VTC Spring), Sydney, NSW, Australia*, pp. 1–6 (2017)

[35] F. Jameel, Faisal, M.A.A. Haider, A.A. Butt, Physical layer security under Rayleigh/Weibull and Hoyt/Weibull fading, in *IEEE ICET, Islamabad, Pakistan*, pp. 1–5 (2017)

[36] Kong L, Kaddoum G (2019). Secrecy characteristics with assistance of mixture Gamma distribution. IEEE Wirel. Commun. Lett..

[37] Pan G, Tang C, Zhang X, Li T, Weng Y, Chen Y (2016). Physical-layer security over non-small-scale fading channels. IEEE Trans. Veh. Technol..

[38] Lei H, Gao C, Guo Y, Pan G (2015). On physical layer security over generalized Gamma fading channels. IEEE Commun. Lett..

[39] Kong L, Tran H, Kaddoum G (2016). Performance analysis of physical layer security over α-μ fading channel. Electron. Lett..

[40] Lei H, Ansari IS, Pan G, Alomair B, Alouini MS (2017). Secrecy capacity analysis over α-μ fading channels. IEEE Commun. Lett..

[41] Kong L, Kaddoum G, Rezki Z (2018). Highly accurate and asymptotic analysis on the SOP over SIMO α-μ fading channels. IEEE Commun. Lett..

[42] Kong L, Vuppala S, Kaddoum G (2018). Secrecy analysis of random MIMO wireless networks over α-μ fading channels. IEEE Trans. Veh. Technol..

[43] Kumar S, Chandrasekaran G, Kalyani S (2015). Analysis of outage probability and capacity for κ-μ/η-μ faded channel. IEEE Commun. Lett..

[44] Bhargav N, Cotton SL, Simmons DE (2016). Secrecy capacity analysis over κ-μ fading channels: Theory and applications. IEEE Trans. Commun..

[45] S. Iwata, T. Ohtsuki, P.Y. Kam, Secure outage probability over κ-μ fading channels, in *IEEE ICC, Paris, France*, pp. 1–6 (2017)

[46] Moualeu JM, Hamouda W (2017). On the secrecy performance analysis of SIMO systems over κ-μ fading channels. IEEE Commun. Lett..

[47] Yang L, Hasna MO, Ansari IS (2018). Physical layer security for TAS/MRC systems with and without co-channel interference over η-μ fading channels. IEEE Trans. Veh. Technol..

[48] Lei H, Gao C, Ansari IS, Guo Y, Pan G, Qaraqe KA (2016). On physical-layer security over SIMO generalized-K fading channels. IEEE Trans. Veh. Technol..

[49] Lei H, Zhang H, Ansari IS, Gao C, Guo Y, Pan G, Qaraqe KA (2016). Performance analysis of physical layer security over generalized-K fading channels using a mixture Gamma distribution. IEEE Commun. Lett..

[50] Lei H, Ansari IS, Gao C, Guo Y, Pan G, Qaraqe KA (2016). Physical-layer security over generalised-K fading channels. IET Commun..

[51] Wu L, Yang L, Chen J, Alouini M (2018). Physical layer security for cooperative relaying over generalized-K fading channels. IEEE Wirel. Commun. Lett..

[52] L. Kong, G. Kaddoum, H. Chergui, On physical layer security over Fox’s H-function wiretap fading channels. IEEE Trans. Veh. Technol. **68**(7), 6608–6621 (2019)

[53] Kong L, Kaddoum G (2018). On physical layer security over the Fisher-Snedecor F wiretap fading channels. IEEE Access.

[54] Badarneh OS, Sofotasios PC, Muhaidat S, Cotton SL, Rabie KM, Aldhahir N (2020). Achievable physical-layer security over composite fading channels. IEEE Access.

[55] Lei H, Luo H, Park KH, Ren Z, Pan G, Alouini MS (2018). Secrecy outage analysis of mixed RF-FSO systems with channel imperfection. IEEE Photon. J..

[56] J.D.V. Sánchez, D.P.M. Osorio, F.J. López-Martínez, M.C.P. Paredes, L. Urquiza-Aguiar, Physical Layer Security of TAS/MRC Over κ-μ Shadowed Fading Channel (2020). arXiv:2005.02441

[57] Ai Y, Kong L, Cheffena M (2019). Secrecy outage analysis of double shadowed Rician channels. Electron. Lett..

[58] Ai Y, Cheffena M, Mathur A, Lei H (2018). On physical layer security of double Rayleigh fading channels for vehicular communications. IEEE Wirel. Commun. Lett..

[59] S.O. Ata, Secrecy performance analysis over double Nakagami-m fading channels, in *IEEE TSP*, pp. 1–4 (2018)

[60] Kong L, Kaddoum G, da Costa DB (2018). Cascaded α-μ fading channels: Reliability and security analysis. IEEE Access.

[61] Tashman DH, Hamouda W, Dayoub I (2020). Secrecy analysis over cascaded κ-μ fading channels with multiple eavesdroppers. IEEE Trans. Veh. Technol..

[62] Moualeu JM, da Costa DB, Hamouda W, Dias US, de Souza RAA (2019). Physical layer security over α-κ-μ and α-η-μ fading channels. IEEE Trans. Veh. Technol..

[63] Chauhan PS, Kumar S, Soni SK (2020). On the physical layer security over Beaulieu-Xie fading channel. AEU-Int. J. Electron. C.

[64] Jia S, Zhang J, Zhao H, Xu Y (2018). Performance analysis of physical layer security over α-η-κ-μ fading channels. China Commun..

[65] Mathur A, Ai Y, Bhatnagar MR, Cheffena M, Ohtsuki T (2018). On physical layer security of α-η-κ-μ fading channels. IEEE Commun. Lett..

[66] Sánchez JDV, Osorio DPM, López-Martínez FJ, Paredes MCP, Urquiza-Aguiar LF (2020). On the secrecy performance over N-wave with diffuse power fading channel. IEEE Trans. Veh. Technol..

[67] R. Singh, M. Rawat, Secrecy capacity of physical layer over κ-μ/Gamma composite fading channel, in *IEEE TENCON*, pp. 1472–1477 (2019)

[68] Al-Hmood H, Al-Raweshidy H (2019). Performance analysis of physical-layer security over fluctuating Beckmann fading channels. IEEE Access.

[69] Jeon H, Kim N, Choi J, Lee H, Ha J (2011). Bounds on secrecy capacity over correlated ergodic fading channels at high SNR. IEEE Trans. Inf. Theory.

[70] Alexandropoulos GC, Peppas KP (2018). Secrecy outage analysis over correlated composite Nakagami-*m*/Gamma fading channels. IEEE Commun. Lett..

[71] Mathur A, Ai Y, Cheffena M, Kaddoum G (2019). Secrecy performance of correlated α-μ fading channels. IEEE Commun. Lett..

[72] Sun J, Bie H, Li X, Zhang J, Pan G, Rabie KM (2019). Secrecy performance analysis of SIMO systems over correlated κ-μ shadowed fading channels. IEEE Access.

[73] Yang L, Liu T, Chen J, Alouini M (2018). Physical-layer security for mixed η-μ and M-distribution dual-hop RF/FSO systems. IEEE Trans. Veh. Technol..

[74] Saber MJ, Sadough SMS (2017). On secure free-space optical communications over Málaga turbulence channels. IEEE Wirel. Commun. Lett..

[75] J. Wang, C. Liu, J. Wang, J. Dai, M. Lin, M. Chen, Secrecy outage probability analysis over Malaga-Malaga fading channels, in *IEEE ICC, Kansas City, MO, USA*, pp. 1–6 (2018)

[76] Lei H, Luo H, Park K, Ansari IS, Lei W, Pan G, Alouini M (2020). On secure mixed RF-FSO systems with TAS and imperfect CSI. IEEE Trans. Commun..

[77] Ai Y, Mathur A, Verma GD, Kong L, Cheffena M (2020). Comprehensive physical layer security analysis of FSO communications over Málaga channels. IEEE Photon. J..

[78] Ai Y, Mathur A, Kong L, Cheffena M (2021). Secure outage analysis of fso communications over arbitrarily correlated Málaga turbulence channels. IEEE Trans. Veh. Technol..

[79] Zeng W, Zhang J, Chen S, Peppas KP, Ai B (2018). Physical layer security over fluctuating two-ray fading channels. IEEE Trans. Veh. Technol..

[80] Zhao H, Yang L, Pan G, Alouini M (2019). Secrecy outage analysis over fluctuating two-ray fading channels. Electron. Lett..

[81] Wang S, Gao Y, Sha N, Zhang G, Zang G (2019). Physical layer security in *K*-tier heterogeneous cellular networks over Nakagami-*m* channel during uplink and downlink phases. IEEE Access.

[82] Ji S, Wang W, Chen H, Zhang S (2019). On physical-layer security of FDA communications over Rayleigh fading channels. IEEE Trans. Cogn. Commun. Netw..

[83] Chun YJ, Cotton SL, Dhillon HS, Lopez-Martinez FJ, Paris JF, Yoo SK (2017). A comprehensive analysis of 5G heterogeneous cellular systems operating over κ-μ shadowed fading channels. IEEE Trans. Wirel. Commun..

[84] Y. Zou, X. Wang, W. Shen, Intercept probability analysis of cooperative wireless networks with best relay selection in the presence of eavesdropping attack, in *IEEE ICC, Budapest, Hungary*, pp. 2183–2187 (2013)

[85] Zou Y, Wang G (2016). Intercept behavior analysis of industrial wireless sensor networks in the presence of eavesdropping attack. IEEE Trans. Ind. Inf..

[86] L. Kong, G. Kaddoum, S. Vuppala, On secrecy analysis for D2D networks over α-μ fading channels with randomly distributed eavesdroppers, in *2018 IEEE ICC Workshop 5G-Security, Kansas City, MO, USA* (2018)

[87] L. Kong, Y. Ai, J. He, N. Rajatheva, G. Kaddoum, Intercept probability analysis over the cascaded Fisher-Snedecor F fading wiretap channels, in *IEEE ISWCS, Oulu, Finland*, pp. 672–676 (2019)

[88] Li J, Petropulu AP (2011). On ergodic secrecy rate for Gaussian MISO wiretap channels. IEEE Trans. Wirel. Commun..

[89] He B, Zhou X (2013). Secure On-Off transmission design with channel estimation errors. IEEE Trans. Inf. Forensics Secur..

[90] L. Kong, G. Kaddoum, D.B. da Costa, E. Bou-Harb, On secrecy bounds of MIMO wiretap channels with ZF detectors, in *IEEE IWCMC, Limassol, Cyprus*, pp. 724–729 (2018)

[91] Liu W, Ding Z, Ratnarajah T, Xue J (2016). On ergodic secrecy capacity of random wireless networks with protected zones. IEEE Trans. Veh. Technol..

[92] Li N, Tao X, Xu J (2014). Ergodic secrecy sum-rate for downlink multiuser MIMO systems with limited CSI feedback. IEEE Commun. Lett..

[93] Frolik J (2007). A case for considering hyper-Rayleigh fading channels. IEEE Trans. Wirel. Commun..

[94] Karas DS, Boulogeorgos AA, Karagiannidis GK (2016). Physical layer security with uncertainty on the location of the eavesdropper. IEEE Wirel. Commun. Lett..

[95] Yoo SK, Cotton SL, Sofotasios PC, Matthaiou M, Valkama M, Karagiannidis GK (2017). The Fisher-Snedecor F distribution: a simple and accurate composite fading model. IEEE Commun. Lett..

[96] Lei H, Dai Z, Ansari IS, Park KH, Pan G, Alouini MS (2017). On secrecy performance of mixed RF-FSO systems. IEEE Photon. J..

[97] Yang L, Jinxia Y, Xie W, Hasna M, Tsiftsis T, Di Renzo M (2020). Secrecy performance analysis of RIS-aided wireless communication systems. IEEE Trans. Veh. Technol..

[98] Kong L, Ai Y, Chatzinotas S, Ottersten B (2021). Effective rate evaluation of RIS-assisted communications using the sums of cascaded α-μ random variates. IEEE Access.

[99] Ai Y, Pereira FA, de Figueiredo R, Kong L, Cheffena M, Chatzinotas S, Ottersten B (2021). Secure vehicular communications through reconfigurable intelligent surfaces. IEEE Trans. Veh. Technol..

[100] Ata SO (2019). Secrecy performance analysis over cascaded fading channels. IET Commun..

[101] Kong L, Chatzinotas S, Ottersten B (2020). Unified framework for secrecy characteristics with mixture of Gaussian (MoG) distribution. IEEE Wirel. Commun. Lett..

[102] Atapattu S, Tellambura C, Jiang H (2011). A mixture Gamma distribution to model the SNR of wireless channels. IEEE Trans. Wirel. Commun..

[103] Al-Hmood H, Al-Raweshidy HS (2017). Unified modeling of composite κ-μ/Gamma, η-μ/Gamma, and α-μ/Gamma fading channels using a mixture Gamma distribution with applications to energy detection. IEEE Antennas Wirel. Propag. Lett..

[104] Selim B, Alhussein O, Muhaidat S, Karagiannidis GK, Liang J (2016). Modeling and analysis of wireless channels via the mixture of gaussian distribution. IEEE Trans. Veh. Technol..

[105] Yilmaz F, Alouini MS (2012). A novel unified expression for the capacity and bit error probability of wireless communication systems over generalized fading channels. IEEE Trans. Commun..

[106] Alhennawi HR, Ayadi MMHE, Ismail MH, Mourad HAM (2016). Closed-form exact and asymptotic expressions for the symbol error rate and capacity of the H-function fading channel. IEEE Trans. Veh. Technol..

[107] Jeong Y, Chong JW, Shin H, Win MZ (2013). Intervehicle communication: Cox-Fox modeling. IEEE J. Sel. Areas Commun..

[108] Prudnikov AP, Brychkov YA, Marichev OI (1990). Integrals and Series: More Special Functions.

[109] Ferdinand NS, da Costa DB, Latva-aho M (2013). Effects of outdated CSI on the secrecy performance of MISO wiretap channels with transmit antenna selection. IEEE Commun. Lett..

[110] Hu J, Yang W, Yang N, Zhou X, Cai Y (2016). On-off-based secure transmission design with outdated channel state information. IEEE Trans. Veh. Technol..

[111] Liu T, Lin S, Hong Y-P (2017). On the role of artificial noise in training and data transmission for secret communications. IEEE Trans. Inf. Forensics Secur..

[112] L. Kong, J. He, G. Kaddoum, S. Vuppala, L. Wang, Secrecy analysis of a MIMO full-duplex active eavesdropper with channel estimation errors, in *IEEE VTC-Fall, Montreal, Quebec, Canada*, pp. 1–5 (2016)

[113] Si J, Li Z, Cheng J, Zhong C (2019). Asymptotic secrecy outage performance for TAS/MRC over correlated Nakagami-*m* fading channels. IEEE Trans. Commun..

[114] Mu P, Li Z, Wang B (2017). Secure on-off transmission in slow fading wiretap channel with imperfect CSI. IEEE Trans. Veh. Technol..

[115] Yao J, Zhou X, Liu Y, Feng S (2018). Secure transmission in linear multihop relaying networks. IEEE Trans. Wirel. Commun..

[116] Si J, Li Z, Cheng J, Zhong C (2019). Secrecy performance of multi-antenna wiretap channels with diversity combining over correlated Rayleigh fading channels. IEEE Trans. Wirel. Commun..

[117] Goel S, Negi R (2008). Guaranteeing secrecy using artificial noise. IEEE Trans. Wirel. Commun..

[118] Wang H, Zheng T, Xia X (2015). Secure MISO wiretap channels with multiantenna passive eavesdropper: artificial noise vs. artificial fast fading. IEEE Trans. Wirel. Commun..

[119] Yan S, Zhou X, Yang N, Abhayapala TD, Swindlehurst AL (2018). Secret channel training to enhance physical layer security with a full-duplex receiver. IEEE Trans. Inf. Forensics Secur..

[120] Jeong C, Kim I, Kim DI (2012). Joint secure beamforming design at the source and the relay for an amplify-and-forward MIMO untrusted relay system. IEEE Trans. Sig. Process..

[121] Wang L, Elkashlan M, Huang J, Tran NH, Duong TQ (2014). Secure transmission with optimal power allocation in untrusted relay networks. IEEE Wirel. Commun. Lett..

[122] Yang N, Yeoh PL, Elkashlan M, Schober R, Yuan J (2013). MIMO wiretap channels: Secure transmission using transmit antenna selection and receive generalized selection combining. IEEE Commun. Lett..

[123] Wang L, Elkashlan M, Huang J, Schober R, Mallik RK (2014). Secure transmission with antenna selection in MIMO Nakagami-*m* fading channels. IEEE Trans. Wirel. Commun..

[124] Yang N, Yeoh PL, Elkashlan M, Schober R, Collings IB (2013). Transmit antenna selection for security enhancement in MIMO wiretap channels. IEEE Trans. Commun..

[125] Zhu J, Zou Y, Wang G, Yao Y-D, Karagiannidis GK (2016). On secrecy performance of antenna-selection-aided MIMO systems against eavesdropping. IEEE Trans. Veh. Technol..

[126] Huang Y, Al-Qahtani FS, Duong TQ, Wang J (2015). Secure transmission in MIMO wiretap channels using general-order transmit antenna selection with outdated CSI. IEEE Trans. Commun..

[127] Moualeu JM, da Costa DB, Lopez-Martinez FJ, Hamouda W, Nkouatchah TMN, Dias US (2019). Transmit antenna selection in secure MIMO systems over α-μ fading channels. IEEE Trans. Commun..

[128] Zheng TX, Wang HM, Yin Q (2014). On transmission secrecy outage of a multi-antenna system with randomly located eavesdroppers. IEEE Commun. Lett..

[129] Vuppala S, Biswas S, Ratnarajah T (2017). Secrecy outage analysis of *k*th best link in random wireless networks. IEEE Trans. Commun..

